# Parallel changes in gene expression in peripheral blood mononuclear cells and the brain after maternal separation in the mouse

**DOI:** 10.1186/1756-0500-2-195

**Published:** 2009-09-25

**Authors:** Johan H van Heerden, Ana Conesa, Dan J Stein, David Montaner, Vivienne Russell, Nicola Illing

**Affiliations:** 1 Department of Molecular and Cell Biology, University of Cape Town, Rondebosch, 7701. South Africa; 2 Bioinformatics Department, Centro de Investigación Principe Felipe, Valencia, Spain; 3 Department of Psychiatry, University of Cape Town, Rondebosch, 7701, South Africa; 4 Department of Human Biology, University of Cape Town, Rondebosch, 7701, South Africa

## Abstract

**Background:**

The functional integration of the neuro-, endocrine- and immune-systems suggests that the transcriptome of white blood cells may reflect neuropsychiatric states, and be used as a non-invasive diagnostic indicator. We used a mouse maternal separation model, a paradigm of early adversity, to test the hypothesis that transcriptional changes in peripheral blood mononuclear cells (PBMCs) are paralleled by specific gene expression changes in prefrontal cortex (PFC), hippocampus (Hic) and hypothalamus (Hyp). Furthermore, we evaluated whether gene expression profiles of PBMCs could be used to predict the separation status of individual animals.

**Findings:**

Microarray gene expression profiles of all three brain regions provided substantial evidence of stress-related neural differences between maternally separated and control animals. For example, changes in expression of genes involved in the glutamatergic and GABAergic systems were identified in the PFC and Hic, supporting a stress-related hyperglutamatergic state within the separated group. The expression of 50 genes selected from the PBMC microarray data provided sufficient information to predict treatment classes with 95% accuracy. Importantly, stress-related transcriptome differences in PBMC populations were paralleled by stress-related gene expression changes in CNS target tissues.

**Conclusion:**

These results confirm that the transcriptional profiles of peripheral immune tissues occur in parallel to changes in the brain and contain sufficient information for the efficient diagnostic prediction of stress-related neural states in mice. Future studies will need to evaluate the relevance of the predictor set of 50 genes within clinical settings, specifically within a context of stress-related disorders.

## Background

The application of microarray techniques has provided insights into the multi-dimensional molecular nature of complex neuropsychiatric disorders. Studies have highlighted the value of using peripheral tissue targets [1,2], an approach based on the functional integration of neural-, endocrine- and immune-systems [3]. Regulatory exchanges between components of these systems provide a foundation for using peripheral tissue targets as indicators of neuropsychiatric states.

One of the earliest demonstrations that gene expression changes in peripheral blood mononucleoctyes (PBMCs) reflected disease states in the brain, was based on a rat model, where acute neural assaults resulted in gene expression changes in PBMCs within 24 hours [4]. Recent studies have focused on human neuropsychiatric disorders with more subtle disruptions in neurophysiology. Segman *et al *[1] were able to predict the onset and progression of post-traumatic stress disorder (PTSD), in recently traumatised patients. Similarly, Tsuang *et al *[2] showed that the microarray analysis of peripheral blood samples discriminated between patients clinically diagnosed with schizophrenia or bipolar disorder and healthy controls. Nevertheless, it remains to be established whether gene expression changes in peripheral tissue targets are paralleled by specific transcriptional alterations in neural tissues [1].

We have used the model of maternal separation, which is known to induce long term alterations in neurophysiology and stress-related behaviours in adult rodents [5,6] to investigate i) whether parallel changes occur in gene expression in three brain regions (the prefrontal cortex, hippocampus, and hypothalamus) and PBMCs and ii) whether gene expression changes in PBMCs could be used to predict the animal treatment group.

## Methods

### Animals and treatment

Maternal separation was carried out on C57BL/6 mice as previously described [6] with some modifications. Briefly, MS litters were separated from dams for 3 h a day, starting at 12 h 00 and ending at 15 h 00, from postnatal day (PND) 1 to 14. SH animals underwent brief daily handling. All subsequent procedures were carried out using males only, as the consequences of separation are gender specific [6].

### Acute restraint stress, sacrifice, blood collection and brain dissections

Mice (N_MS _= 30, N_SH _= 30) were subjected to 10 min of acute restraint stress and allowed to recover for 20 min prior to sacrifice. Restraint stress was chosen as a means of acutely activating the Hypothalamic-Pituitary-Adrenal (HPA) axis (HPAA), which allowed for an assessment of possible differences in plasma corticosterone profiles (van Heerden et al, submitted manuscript). All mice were sacrificed, by means of cervical dislocation, immediately followed by decapitation and collection of trunk blood. Neural tissues: the (1) prefrontal cortex (PFC), (2) hippocampus (Hic) and (3) hypothalamus (HYP) were immediately dissected and submerged in RNALater^® ^(Qiagen Inc., USA).

### Microarray processing and data analysis

Fifty-five samples, 15× PFC (8× MS and 7× SH), 10× Hic and 10× Hyp (5× MS and 5× SH, each and 20× PBMC (10× MS and 10× SH) were used for microarray processing, with a two-colour common reference design. Samples were matched, so that 10 individuals (5× MS and 5× SH) were completely represented in all tissues. A common reference pool was constructed by combining equal amounts (0.75 μg) of PFC and Hic RNA from both groups. Commercial pre-spotted, full mouse genome, microarray slides (OpArray™) were sourced from Operon (Operon Biotechnologies, Germany). Full details of RNA labelling, microarray hybridization, image capture and microarray data processing are given in Additional file 1: Supplementary Methods. Microarray data are available in the ArrayExpress database  under accession number E-MEXP-2101.

Data normalization was done in R, using the Limma package [7]. Pre-processing and removal of batch effects were done using GEPAS  and ASCA-genes [8] respectively. Differentially expressed genes were identified using a concordance strategy [9], based on overlap between three statistically divergent approaches. Genes that had a P-value < 0.05, using both the Info statistic, from the ScoreGenes software package , and the Tusher *et al *[10] Significance Analysis of Microarrays (SAM) implementation in the T-Rex module of GEPAS , in addition to an absolute fold-change > 1.2 (where fold change is defined as the fold difference between MS and SH), were considered to be differentially expressed (DE).

All data clustering was done in the Tigr MultiExperiment Viewer V4.1 (TMEV, ) using a Pearson correlation metric with average linkage. Functional enrichment of GO terms within differentially expressed gene sets was evaluated using Blast2GO [11]. Gene set enrichment analysis on lists ordered according to SAM statistics was done using FatiScan [12]. The PFC and Hyp lists were evaluated using 50 partitions, the PBMC list using 55 partitions and the Hic list using 60 partitions.

The efficiency of PBMC gene expression profiles at predicting the treatment class of samples (i.e. MS or SH) was evaluated with the Prophet module in GEPAS [13] using both the K-nearest neighbour (KNN) and Support Vector machine (SVM) algorithm options. Leave-one-out cross validation was used to counter selection bias whilst simultaneously assessing prediction efficacy.

## Results and Discussion

Microarray data comparing the response of control and MS adult mice to stress was used to investigate the presence of a functional link between gene expression changes in the brain and PBMCs. In the first instance data was analysed to characterise the transcriptional response of three brain regions, the prefrontal cortex, the hippocampus and hypothalamus to stress, and to investigate whether a co-ordinated change in glutamatergic and GABAergic systems occurred in MS mice. Corresponding differences in gene expression in PBMCs of MS mice compared to control mice were also identified. Importantly, these differences could be used to predict the treatment status of mice.

### Microarray analysis

After normalization, replicate merging, removal of flagged features and imputation, the number of genes expressed in each tissue was: (1) PFC, 15 760; (2) Hic, 17 344; (3) Hyp, 15 794 and (4) PBMC, 13 306.

### MS produced gene expression differences in all tissues

**Figure 1:**
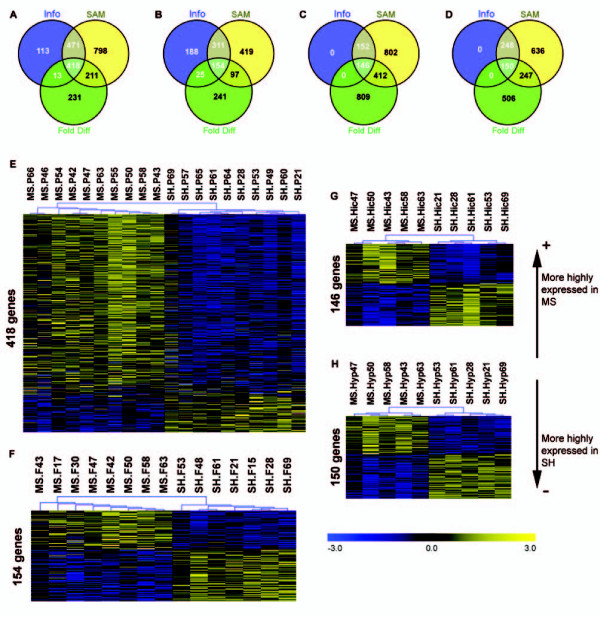
**Differential gene expression results**. Venn diagrams show the overlap between different gene selection criteria (Info and SAM P < 0.05 and Fold difference > 1.2) for (A) PBMC, (B) pFC, (C) Hic and (D) Hyp. This gene selection strategy significantly reduced the number of genes identified as DE by any one single criterion. Also shown are the false colour sample profiles of hierarchically clustered differentially expressed genes for (E) PBMC samples [347 over- and 71 under-expressed], and neural tissues (F) pFC [66 over- and 88 under-expressed], (G) Hic [71 over- and 75 under-expressed] and (H) Hyp [69 over- and 81 under-expressed]. The selected genes produce a clear separation between MS and SH samples. Genes more highly expressed in MS samples are at the top and those more highly expressed in SH samples at the bottom. P = PBMC; F = pFC.

Differentially expressed (DE) genes were identified in all tissues (Figure 1A-D). A summary of all DE genes is provided in [see Additional file 2 Table S2], [see Additional file 3 Table S3], [see Additional file 4 Table S4], and [see Additional file 5 Table S5]. The unsupervised hierarchical sample clustering of differentially expressed genes, produced clear group (MS or SH) separations within all tissues (Figure 1E-H). No single gene was differentially expressed across all tissues.

### Gene set enrichment analysis revealed significant functional themes

**Figure 2:**
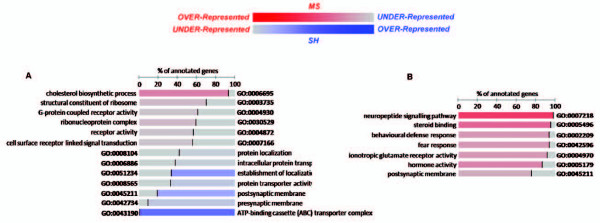
**FatiScan gene set enrichment results**. Shown are significant co-ordinately expressed GO terms within whole gene sets for (A) PFC and (B) Hic. The normalized percentage of genes annotated with a specific term is indicated for each group. Red indicates coordinated over-expression in MS group and Blue coordinated over-expression SH group (or under-expression in MS group). Colour intensity denotes how strongly a term is over- or under-expressed.

**Figure 3:**
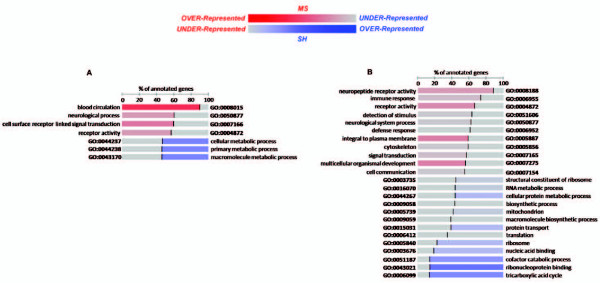
**FatiScan gene set enrichment results**. Shown are significant co-ordinately expressed GO terms within whole gene sets for (A) Hyp and (B) PBMC. The normalized percentage of genes annotated with a specific term is indicated for each group. Red indicates coordinated over-expression in MS group and Blue coordinated over-expression SH group (or under-expression in MS group). Colour intensity denotes how strongly a term is over- or under-expressed.

The FatiScan analysis revealed the significant enrichment of functional terms, in all tissues (Figure 2 and Figure 3). In PBMC samples (Figure 3B), over-expressed terms could be grouped, generally, into signalling- (GO:0004872, GO:0051606, GO:0005887, GO:0007165, GO:0007154), immune- (GO:0006955, GO:0006952, GO:0005856, GO:0007275) and, interestingly, neurologically-related (GO:0008188, GO:0050877) classes. On the other hand, under-expressed terms all displayed a metabolic theme, with terms related to RNA and protein processing (GO:0003735, GO:0016070, GO:0044267, GO:0009058, GO:0009059, GO:0015031, GO:0006412, GO:0005840, GO:0003676 and GO:0043021) and energy metabolism (GO:0005739, GO:0051187 and GO:0006099). These results suggest a functional shift in the immune system in PBMCs in MS mice, characterised by the coordinated down-regulation of energy requiring processes, such as protein synthesis and transport. This functional shift might reflect the well characterised mobilisation of energy and inhibition of further storage in response to stress [14].

### Response of the glutamergic and GABergic systems in neural tissues after stress

DE genes and enriched functional terms from the PFC datasets highlighted the importance of the glutamatergic and GABAergic systems in the stress-related response of the MS mice. These two neurotransmitter systems constitute the major stimulatory (glutamate) and inhibitory (GABA) mechanisms of neurotransmission, and work counteractively to ensure optimal neuronal activity after stress [15]. Glutamatergic signalling was enhanced in MS mice possibly as a consequence of deficiencies in GABAergic mediated inhibitory mechanisms.

**Figure 4:**
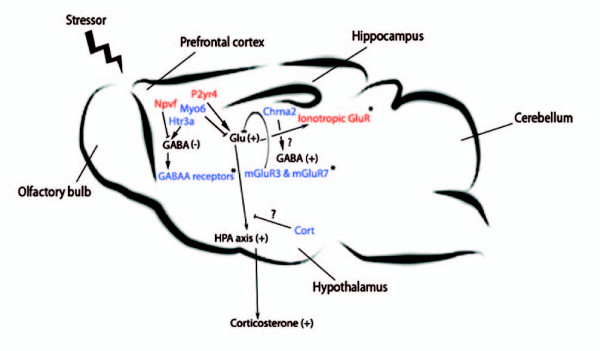
**Schematic summary of neural gene expression results in support of a stress-related hyperglutamatergic state in MS brain samples**. Such a hyperglutamaterigc state could potentially result in elevated stress-induced corticosterone responses. Red indicates over-expression and blue under-expression, in MS samples, respectively. An asterisk indicates genes or functional classes that were found to be regulated in a coordinated manner. Glu = Glutamate, (+) indicates increased signalling activity, (-) indicates decreased signalling/acitivity.

DE genes whose products are involved in the modulation of glutamatergic and GABAergic signalling included *P2yr4 *and *Npvf *(Figure 4). The activation of P2yr4 positively regulates glutamate release [16], whereas Npvf is an important inhibitor of GABAergic neurotransmission [17]. The over-expression of both these genes in the MS PFC tissue, points to a hyperactive glutamatergic system. Supporting this observation is the under-expression of *Myo6 *in the MS samples. Myo6 is crucial for the efficient endocytosis of postsynaptic glutamate receptors, with deficiencies resulting in increased excitatory neurotransmission [18]. *Htr3a *was also under-expressed in MS samples. This receptor is strongly associated with GABAergic neurons and interneurons which activate the GABA mediated inhibitory neurotransmission in the prefrontal cortex [19]. The co-ordinated under-expression of both pre- and post-synaptic component GO terms further supports the hypothesis of a hyperglutamatergic state in the PFC of MS mice (Figure 4). Specifically, genes supporting depletion of postsynaptic components in MS mice included three GABA_A _receptors (GABA_A _alpha-1 and -3, and GABA_A _gamma-3) (Figure 4); such receptors mediate inhibition of neurotransmission with disruptions resulting in enhanced anxiety [20]. Genes supporting functional depletion of presynaptic components included two metabotropic glutamate receptors, *mGluR3 *and *mGluR7 *(Figure 4). These receptors participate in negative feedback mechanisms that inhibit presynaptic glutamate release. Results from the hippocampal gene expression dataset extend these observations, with the over-representation, in MS samples, of genes involved in ionotropic glutamate signalling (Figure 4). Although this hyperglutamatergic theme was not readily apparent in either the DE genes or functionally enriched terms of the hypothalamus dataset, under-expression of cortistatin may be relevant insofar as cortistatin signalling inhibits glutamate induced responses in hypothalamus [21] (Figure 4).

These findings are consistent with the central role of glutamate in the stress-response, in structures such as PFC and hippocampus. Stressors such as acute restraint have been shown to produce dramatic and rapid increases in glutamate levels primarily in the PFC, which ultimately culminates in HPAA activation and glucocorticoid secretion. In addition, the hippocampus is a major site of stress-associated glutamate action. The mechanisms which regulate glutamate action and release within this region function downstream of prefrontal cortical processes, constituting a secondary stress-response phase, which, unlike the PFC, is sensitive to neuroendocrine modulation [22]. The glutamatergic signature found here in both the PFC and hippocampus is therefore consistent with previous work.

### Functional significance of gene expression changes in PBMC tissues

A large number of genes (418) were found to be differentially expressed between MS and SH individuals and included several genes whose products are important modulators of immune system function. Examples include *Foxp3*, an essential modulator of T cell function [23]; *IL-17ra*, the receptor target for the IL-17 mediated inflammatory pathway [24]; and *Ccl5 *(also known as *Rantes*), which regulates the activity of several cellular populations within the immune system [25].

The evidence obtained from the neural transcriptomes (combined with corticosterone and behavioural profiles; van Heerden et al Submitted Manuscript) indicates that pre-weaning treatment (MS or SH) result in differential stress-related profiles. Given this context, the gene expression information derived from the PBMC samples was evaluated in terms of its ability to derive accurate predictions of pre-weaning status of individuals.

### PBMC gene expression profiles accurately predict sample classes

**Figure 5:**
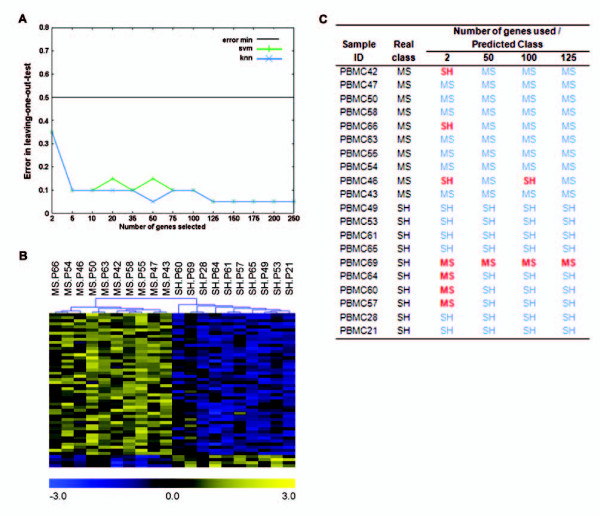
**Sample classification and prediction results**. (A) Leave-one-out error rates of classifiers. The KNN algorithm (blue line) reaches an optimal prediction efficiency of 95% with a minimum of 50 genes. Using 125 genes the SVM algorithm (green line) obtains this efficiency, and converges with KNN. (B) Hierarchically sample clustered (Pearson correlation metric with average linkage) profiles for the 50 gene predictor set. Notice, that although only 19 out of 20 samples were correctly classified, hierarchical clustering separates all samples into two general treatment-related clusters. (C) A summary of KNN sample classification results, showing details of the misclassification of individual samples. Although most samples classes were correctly predicted, PBMC69, an SH sample, was consistently misclassified. P = PBMC.

The classification and prediction of sample classes (MS or SH) using PBMC gene expression values, were found to be highly efficient. Using KNN (with 4 neighbours), 50 genes (Figure 5; Table 1) were sufficient to accurately identify sample classes 19 out of 20 times. Most of the genes included in the predictor were over-expressed (Figure 5B). SVM, however, only achieved this success rate using a minimum of 125 genes (with linear and radial kernels). Importantly, this 125 gene set consisted of the 50 genes included in Table 1, in addition to 75 other genes, which were the same for both algorithms (data not shown).

**Table 1:** Summary of 50 gene predictor set, which classified samples with 95% accuracy*

**Operon Oligo ID**	**Description**	**Symbol**	**ENSEMBL/Refseq/Riken ID**	**Over/Under expressed in MS**
M400008627	RIKEN cDNA 4921528I07 gene	*4921528I07Rik*	ENSMUSG00000074149	over
M200012683	Acetyl-Coenzyme A acetyltransferase 2	*Acat2*	ENSMUSG00000023832	over
M400004596	A disintegrin-like and metalloprotease with thrombospondin type 1 motif, 9	*Adamts9*	ENSMUSG00000030022	over
M200000582	Adenylate cyclase 8	*Adcy8*	ENSMUSG00000022376	over
M200005645	Actin related protein 2/3 complex, subunit 5-like	*Arpc5l*	ENSMUSG00000026755	over
M200006901	ATPase, H+ transporting, lysosomal V0 subunit E2	*Atp6v0e2*	ENSMUSG00000039347	over
M400004024	cDNA sequence BC013672	*BC013672*	ENSMUSG00000037921	over
M400008030	Bone gamma-carboxyglutamate protein, related sequence 1	*Bglap-rs1*	ENSMUSG00000074489	over
M300011602	Carbonic anhydrase 14	*Car14*	ENSMUSG00000038526	over
M200000995	Cholecystokinins precursor	*Cck*^§^	ENSMUSG00000032532	over
M200013753	Coronin 7	*Coro7*	ENSMUSG00000039637	over
M200003934	Cytochrome P450, family 2, subfamily c, polypeptide 29	*Cyp2c29*	ENSMUSG00000003053	over
M300013894	RIKEN cDNA D130054N24 gene	*D130054N24Rik*	ENSMUSG00000042790	over
M400003995	RIKEN cDNA D330050I23 gene	*D330050I23Rik*	ENSMUSG00000072569	over
M300010488	Dermokine	*Dmkn*	ENSMUSG00000060962	over
M200003607	Dedicator of cytokinesis 7	*Dock7*	ENSMUSG00000028556	over
M300014949	Endothelial differentiation, sphingolipid G-protein-coupled receptor, 5	*Edg5*	ENSMUSG00000043895	over
M400001692	Predicted gene	*EG620592*	ENSMUSG00000071719	over
M400010593	Forkhead box protein R1 (Forkhead box protein N5)	*Foxr1*	ENSMUSG00000074397	over
M300000132	Homeo box A4	*Hoxa4*	ENSMUSG00000000942	over
M400013298	LSM14 protein homolog A (Rap55)	*Lsm14a*	ENSMUSG00000066568	over
M400004821	Lysocardiolipin acyltransferase	*Lycat*	ENSMUSG00000054469	over
M400009939	Mitogen-activated protein kinase kinase kinase 9	*Map3k9*	ENSMUSG00000042724	over
M300007290	Mesoderm posterior 2	*Mesp2*	ENSMUSG00000030543	over
M200007123	Muted protein	*Muted*^§^	ENSMUSG00000038982	under
M200010626	Matrix-remodelling associated 8	*Mxra8*	ENSMUSG00000073679	over
M200007448	Nitric oxide synthase interacting protein	*Nosip*	ENSMUSG00000003421	over
M300018063	Olfactory receptor 1495	*Olfr1495*	ENSMUSG00000047207	over
M300017588	Olfactory receptor 66	*Olfr66*	ENSMUSG00000058200	over
M300015973	Olfactory receptor 669	*Olfr669*	ENSMUSG00000073916	over
M300002331	Predicted gene	*MGI:3652048*	ENSMUSG00000020682	over
M200003458	Oxytocin	*Oxt*	ENSMUSG00000027301	over
M400010890	Mus musculus polymerase (RNA) II (DNA directed) polypeptide C	*Polr2c*	ENSMUSG00000031783	over
M200000936	Peripherin 1	*Prph1*	ENSMUSG00000023484	over
M300003403	PTK2 protein tyrosine kinase 2	*Ptk2*	ENSMUSG00000022607	under
M400001722	Slingshot homolog 3 (Drosophila)	*Ssh3*	ENSMUSG00000034616	over
M300003482	Type 2 lactosamine alpha-2,3-sialyltransferase	*St3gal6*^§^	ENSMUSG00000022747	under
M200000227	Stromal interaction molecule 1	*Stim1*	ENSMUSG00000030987	over
M300001453	Surfeit gene 5	*Surf5*	ENSMUSG00000015776	over
M400000616	Thrombopoietin precursor	*Thpo*	ENSMUSG00000022847	over
M400009774	Transmembrane BAX inhibitor motif containing 1	*Tmbim1*	ENSMUSG00000006301	over
M200013582	Transmembrane protein 25	*Tmem25*	ENSMUSG00000002032	over
M400000938	Transmembrane protein 63A	*Tmem63a*^§^	ENSMUSG00000026519	under
M400013169	Xin actin-binding repeat containing 2 isoform 2	*Xirp2*	ENSMUSG00000027022	over
M400014435	Zinc finger protein 84	*Zfp84*	ENSMUSG00000046185	over
M400018008	Novel Protein	*Not assigned*	AC160535	over
M400012711	Novel protein (I830077J02Rik)	*Not assigned*	AC121847	over
M400017112	Uncharacterised	*Not assigned*	AK054246	over
M400003712	Uncharacterised	*Not assigned*	AC122270	over
M400008575	Uncharacterised	*Not assigned*	ENSMUSG00000064159	over

*Genes are sorted by gene symbol; § Not included in differentially expressed gene list

Of the 50 genes included in the predictor, 46 were functionally annotated. Of particular interest was the identification of 3 genes, *Oxt*, *Cck *and *Adcy8 *(all over-expressed), whose products are known to be important mediators of stress- and anxiety-associated behaviours (Table 1) [26-28]. Both Oxt and Cck are neuroactive hormones with previously described endogenous immunomodulatory properties [29,30]. These results confirm that the transcriptional profiles of peripheral immune tissues do indeed contain sufficient information for the efficient diagnostic prediction of stress-related neural states in mice. Products of these genes may participate in pathways that are particularly sensitive to stress-induced regulation of the immune system.

## Competing interests

The authors declare that they have no competing interests.

## Authors' contributions

JHvH carried out the animal studies, microarray experiments, data analysis and drafted the manuscript. NI designed and supervised the study, and assisted with the writing of the manuscript. DM and AC assisted with the analysis of the microarray data. DJS and VR contributed to the conception and design of the study, and assisted in the editing of the final versions of the manuscript. All the authors read and approved the final manuscript.

## Supplementary Material

Additional file 1 **Supplementary Methods**. Detailed description of materials and methods, including a summary of RNA sample purity and integrity, and examples of box- and MA-plots from the PFC microarray dataset. Click here for file

Additional file 2 **Table S2 Frontal Association Cortex differentially expressed genes**. Summary of differentially expressed genes identified in frontal association cortex, including p-values for Info and SAM statistics, and log_2 _fold differences. Click here for file

Additional file 3 **Table S3 Hippocampus differentially expressed genes**. Summary of differentially expressed genes identified in hippocampus, including p-values for Info and SAM statistics, and log_2 _fold differences. Click here for file

Additional file 4 **Table S4 Hypothalamus differentially expressed genes**. Summary of differentially expressed genes identified in hypothalamus, including p-values for Info and SAM statistics, and log_2 _fold differences. Click here for file

Additional file 5 **Table S5 Peripheral Blood Mononuclear Cells differentially expressed genes**. Summary of differentially expressed genes identified in Peripheral Blood Mononuclear Cells, including p-values for Info and SAM statistics, and log_2 _fold differences. Click here for file
