# Supplementary material for: Parallel changes in gene expression in peripheral blood mononuclear cells and the brain after maternal separation in the mouse
Source: BMC Res Notes. 2009 Sep 25;2:195. doi: 10.1186/1756-0500-2-195 (PMC2759952; doi:10.1186/1756-0500-2-195)
Supplement: Additional file 1 — Supplementary Methods. Detailed description of materials and methods, including a summary of RNA sample purity and integrity, and examples of box- and MA-plots from the PFC microarray dataset. [file 1756-0500-2-195-S1.DOC]

**Supplementary Methods**

**Animals and Maternal Separation procedure**

The protocol was approved by the animal ethics committee of the University of Cape Town (Ethics clearance number: 006/007).

Postnatal day (PND) 0 was assigned to litters born before 15h30 each day. Litters were randomly assigned to undergo maternal separation (MS; n = 10) or to be reared under standard conditions, with simulated handling (control) events (SH; n = 9). The average litter size of both MS and SH groups was equal (n = 7).

All animals were maintained under a 12 h light-dark cycle (lights on from 6h00 to 18h00). Temperature was kept at 21 ± 2 °C. Animals had ad libitum access to sterilized food and tap water. All animal-human interactions were limited to a single researcher.

The MS dams were first removed from the home cage, after which the pups were moved to a clean cage, which was kept at the ambient temperature of the vivarium. The dam was placed back in the home cage and moved to a separate room for the duration of the separation, this to exclude olfactory or ultrasound vocalization exchanges between dams and their pups. After 3 h, pups and dams were reunited in their home cage. SH animals underwent daily handling. SH dams were removed from the home cage; pups were briefly moved to a clean cage and immediately returned to their home cage, followed by the dam. This procedure simulated the handling undergone by MS pups and served as a control, never lasting for more than 5 min per litter. At PND 21, all pups were weaned and group housed by sex and treatment.

**PBMC isolation**

After collection of trunk blood, 250 μl aliquots were added to 12 ml sterile test tubes (Bibby Sterilin Ltd., UK), followed by 5 ml of a prepared tricine-buffered-saline(TBS)-Iodixanol mixture (TBS: 0.85% NaCl, 10 mM Tricine, pH 7.4; TBS-iodixanol: 5 ml TBS and 1.5 ml Optiprep™). After mixing, an additional 0.5 ml TBS was gently layered on top of the blood-TBS-Optiprep™ mixture. Samples were centrifuged at 1000 g for 30 min at room temperature. PBMCs were collected, from the meniscus downward, in 4 ml of medium and added to a clean 12 ml tube. This suspension was diluted with two volumes of TBS. Cells were pelleted at 400 g for 10 min. The supernatant was carefully decanted, cells snap frozen in liquid N_2_, and stored at -80°C until further processing.

***RNA sample processing***

RNA extracts were quantitated and the purity (A_260_/A_280_ nm) assessed using the Nanodrop ND-1000 spectrophotometer system (Nanodrop Technologies, USA). RNA integrity was determined using the Agilent BioAnalyzer 2100 System (Agilent, USA). A summary of sample purity (A_260_/A_280_ nm ratios) and sample integrity measures, reported as a RIN, is provided in Table S1. RIN numbers for PBMC microarray samples were not generated, as total RNA obtained from these samples was critically limited. Instead, a surrogate sample set, representing total RNA from PBMC samples which were processed in parallel, with suboptimal purity measures (A_260_/A_280_ nm ratios < 1.7) and which could not be matched to samples from other tissues, was used to determine the general integrity of RNA obtained from PBMC samples.

**Table S1** Summary of microarray sample purity and integrity

| **Tissue / Measure** | **pFC** | | **Hic** | | **Hyp** | | **PBMC** | | **Surrogate* PBMC** | |
| --- | --- | --- | --- | --- | --- | --- | --- | --- | --- | --- |
|  | **Mean** | **S.D.** | **Mean** | **S.D.** | **Mean** | **S.D.** | **Mean** | **S.D.** | **Mean** | **S.D.** |
| **A_260_/A_280_** | 2.07 | 0.04 | 2.01 | 0.04 | 2.1 | 0.01 | 1.90 | 0.15 | 1.62 | 0.07 |
| **RIN** | 7.00 | 1.14 | 5.7 | 0.71 | 6.2 | 0.33 | N/A | N/A | 7.8 | 0.91 |

*****A set of PBMC samples with suboptimal A_260_/A_280_ nm ratios (<1.7), which could not be matched with other samples from other tissues and which was used to infer PBMC sample integrity for parallel processed samples. S.D. = Standard Deviation

Although Hic and Hyp RINs were low, all samples were efficiently amplified, which indicated intact 3`-ends as the amplification method required the presence of intact poly(A)-tails (see Ambion Amino Allyl MessageAmp™ II method (http://www.ambion.com/techlib/prot/fm_1753.pdf). Selection of PBMC samples used for microarray hybridisation was also based on post-amplification RNA profiles (data not shown).

**Labelling and hybridization**

All labelling reactions were carried out using 6.5 μg of amplified RNA (antisense RNA, aRNA). Reference aRNA was labelled with Cy5 and sample aRNA with Cy3™. Labelled samples were hybridized to OpArray^TM^ slides (Operon Biotechnologies, Germany), which were printed with version 4.0 of the Mouse Genome Oligo Set representing ± 25,000 mouse genes and approximately 38,000 gene transcripts. The slides were prepared and processed according to the manufacturer’s instructions. Briefly, hybridisations were performed using 170 ng of Cy3^TM^ labelled sample and Cy5 labelled reference, at 42°C for 16 h in humidified ArrayIt® hybridisation chambers (Telechem, USA). After washing, slides were dried by centrifugation at 200 g for 5 min. Slides were kept in a light protected air tight environment and scanned on the same day.

***Image acquisition***

All images were acquired using an Axon 4000A dual-colour confocal laser scanner coupled to Genepix 6.0.27 Pro Software (Axon Instruments / Molecular Devices Corporation, CA, USA). Fluorescent signals were collected in Cy3 and Cy5 channels and quantified using automatic morphological feature alignment and background estimation with manual adjustments where necessary. A predefined filter was used to flag features that failed to meet a set of minimum quality criteria. The criteria were as follows:

[Name] = "Dye Marker" And

[Flags] = [Clear] Or

[Name] = "Buffer" And

[Flags] = [Clear] Or

[Name] = "null" And

[Flags] = [Clear] Or

[Name] = "EMPTY" And

[Flags] = [Clear] Or

[F Pixels] < 25 And

[Flags] = [Clear] Or

[F532 Median] > 65532 Or

[F635 Median] > 65532 Or

[F532 CV] < 100 And

[F532 Median] < 50 And

[Flags] = [Clear] Or

[F635 CV] < 100 And

[F635 Median] < 50 And

[Flags] = [Clear]

**Pre- and post-normalization data quality**

Data quality, pre- and post-normalization, was carefully assessed using common visualization techniques such as boxplots and MA-plots. As an example, data from the PFC set is used to illustrate the general method (Additional figs. 1 and 2).

Based on these evaluations the appropriate normalization strategy was chosen for each data set. E.g. for the PFC data set (this example) Global loess + Between array scaling (Figs. 1 & 2 C) was chosen as the most optimal normalization strategy. This method resulted in the symmetrically distributed data (which is one of the expectations of appropriately normalized whole genome datasets), whilst maintaining a good measure of variability (i.e. no over-compression of data, which often results in the artificial removal of noise – see Fig. 2D)

***Pre-processing and removal of batch effects***

Duplicate feature values were merged and missing values imputed using the Pre-processing module of GEPAS ([1]; [http://www.gepas.org](http://www.gepas.org/)). A first round of duplicate merging was done based on unique oligo identifiers (i.e. all features with the same oligo sequence). Following missing value imputation, a second round of duplicate merging was performed using primary gene identifiers (ENSEMBL Mouse release 43.36d, [http://www.ensembl.org](http://www.ensembl.org/); Refseq release 22, [http://www.ncbi.nlm.nih.gov](http://www.ncbi.nlm.nih.gov/); or Riken release 3.0, [http://fantom.gsc.riken.jp](http://fantom.gsc.riken.jp/); where available and in order of preference), thus reducing the number of duplicate gene measurements. KNN imputation (with the default of 15 nearest neighbours) was used to estimate missing values for (1) PFC patterns with a minimum of 66% unflagged features (i.e. at least 10 out of 15 slides), (2) Hic and Hyp patterns with a minimum of 70% unflagged features (i.e. at least 7 out of 10 slides) and (3) PBMC patterns with a minimum of 55% unflagged features (i.e. 11 out of 20 slides).

Batch effects and other forms of structured noise were removed from data using ASCA-genes [2]. In the current study, a batch was defined as a single amplification, labelling, hybridisation and scanning run, which included five slides. Other sources of structured noise were identified as residual error and removed if any one factor accounted for more than four times the error that would be expected by chance.

**References**

1. Montaner D, Tárraga J, Huerta-Cepas J, Burguet J, Vaquerizas JM, Conde L, Minguez P, Vera J, Mukherjee S, Valls J et al: Next station in microarray data analysis: GEPAS. Nucleic Acids Res 2006, 34(Web Server issue).

1. Nueda M, Conesa, A., Westerhuis, J., Hoefsloot, H., Smilde, A., Talon, M. and Ferrer, A.: Discovering gene expression patterns in time course microarray experiments by ANAVO-SCA. Bioinformatics 2007, 23(14):8.

Additional Figures

| 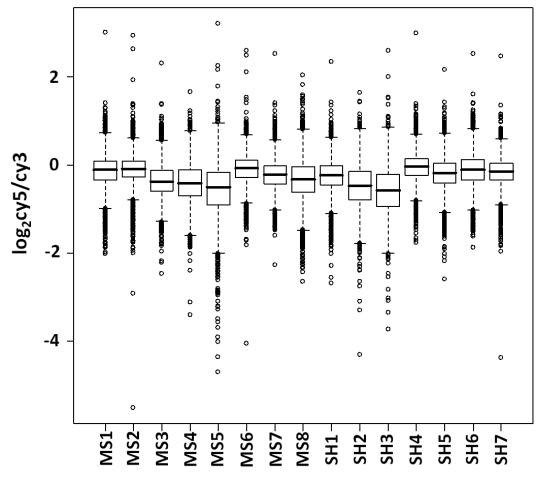  **A**  **B** | 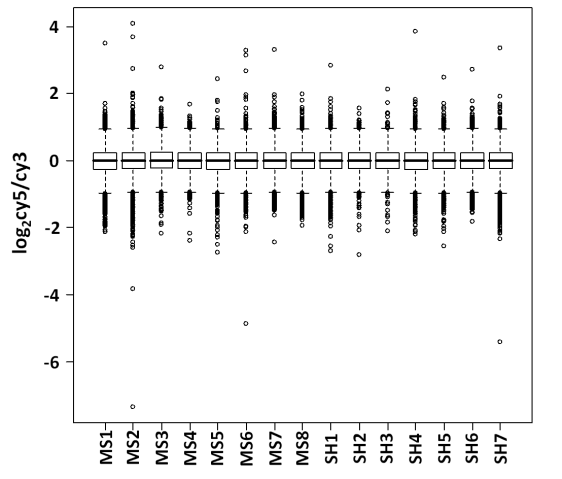  **C** | 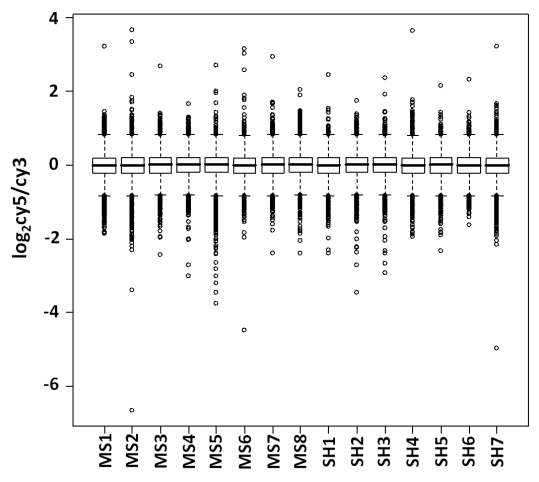 | 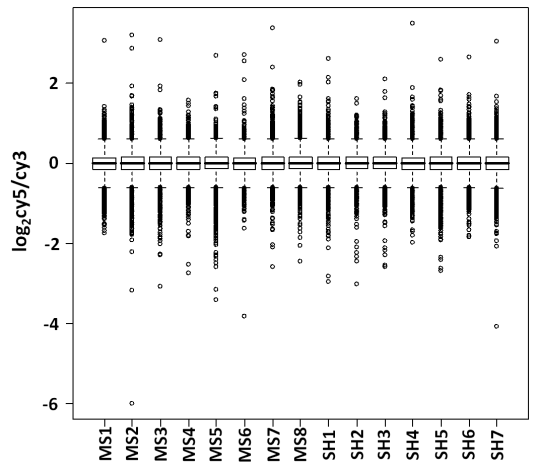  **D** |
| --- | --- | --- | --- |

**Additional Figure 1: Boxplot summaries of log_2_ expression ratios (Cy5/Cy3) from the PFC data set.** Boxplot summaries for (A) prenormalized data, highlight differences in the data distributions between arrays. These distributions should exhibit similar profiles, regardless of treatment. Post-normalization profiles are shown for (B) Median normalization + between array scaling, (C) Global Loess + between array scaling and (D) Print-tip Loess + between array scaling. An assessment of these images suggests that all three methods have successfully normalized the data. The level of resolution provided by these profiles make a distinction between the efficacies of different methods difficult; therefore these evaluations were combined with MA-plots (see below)

| **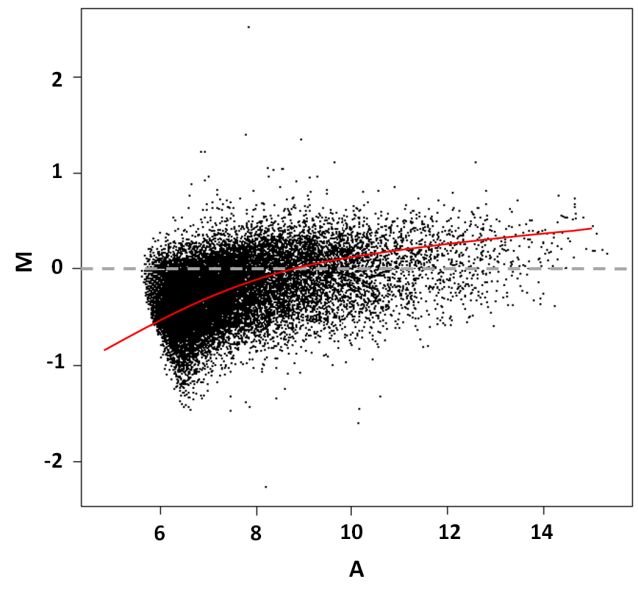**  **A**  **B** | **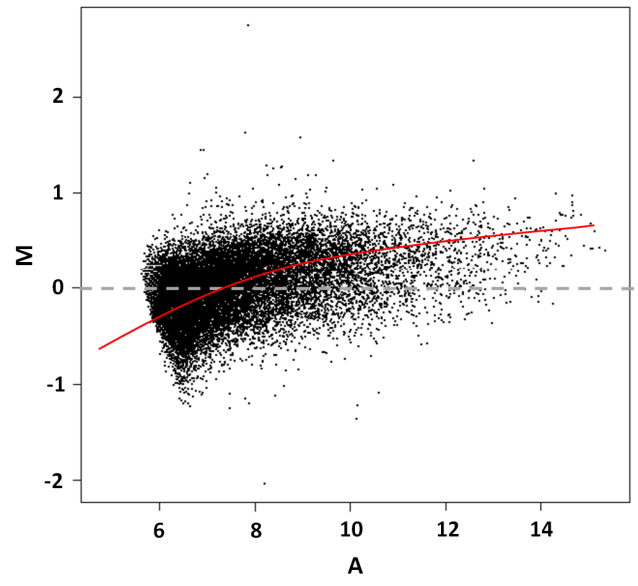** |
| --- | --- |
| **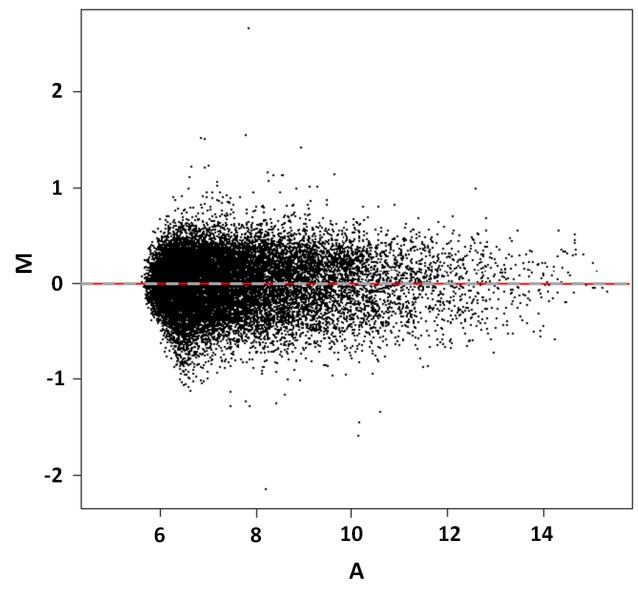**  **D**  **C** | **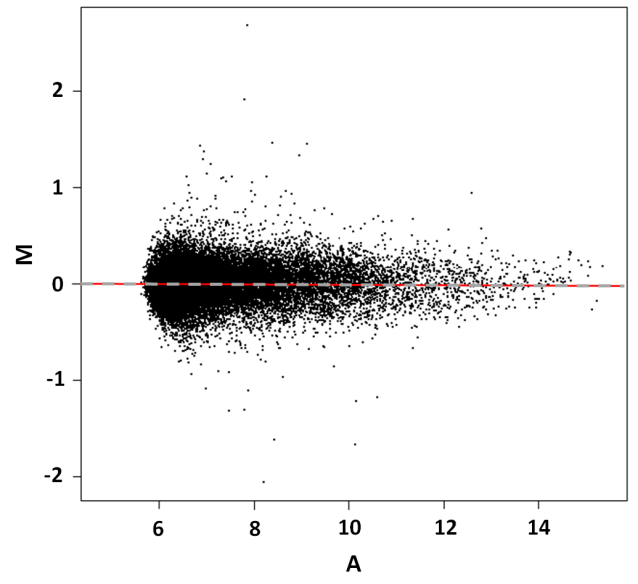** |

**Additional Figure 2: Pre- and post-normalization MA-plots of a representative pFC array.** Shown here are MA-pots for (A) pre-normalized data, in addition to (B) Median, (C) Global Loess and (D) Print-tip Loess normalized data (for a representative slide). The grey dashed-horizontal line indicates the zero ratio axis, around which the data is expected to be symmetrically distributed. The red line illustrates the general trend in the data. In (A) this trend highlights the presence of a non-linear ratio-intensity bias, which is not corrected by (B) Median normalization. In contrast, both Global (C) and Print-tip Loess (D) methods effectively removed this non-linear trend, resulting in data symmetrically distributed around the zero horizontal. Also note, that Print-tip Loess normalization (D) appears to compress the data, relative to the other methods. M = log ratio = (log_2_Cy5/Cy3); A = average intensity = ½Log_2_[(Cy5 intensity) + (Cy3 intensity)].
